# Abnormal spindle-like microcephaly-associated protein (ASPM) contributes to the progression of Lung Squamous Cell Carcinoma (LSCC) by regulating CDK4

**DOI:** 10.7150/jca.39760

**Published:** 2020-07-11

**Authors:** Ya-Jing Yuan, Yao Sun, Rong Gao, Zhen-zhen Yin, Zhi-yong Yuan, Li-Ming Xu

**Affiliations:** 1 Department of Anesthesia, Tianjin medical university cancer institute & hospital, National clinical research center for cancer, Key laboratory of cancer prevention and therapy, Tianjin's Clinical Research Center for Cancer, Tianjin, 300060, China.; 2 Department of Radiation Oncology, Tianjin medical university cancer institute & hospital, National clinical research center for cancer, Key laboratory of Cancer Prevention and Therapy, Tianjin's clinical research center for cancer, Tianjin, 300060, China.; 3 Department of Pathology, Gansu Medical College, Pingliang City, Gansu Province, 744000, China.; 4 Department of Radiation Oncology, Tianjin Medical University Cancer Hospital airport hospital, Tianjin, 300308, China.

**Keywords:** Lung squamous cell carcinoma, Proliferation, ASPM, CDK4, Therapeutic target

## Abstract

Lung cancer is a type of malignant tumor with high morbidity and mortality. Due to its complicated etiology and clinical manifestations, no significant therapeutic advance has been made. Lung squamous cell carcinoma (LSCC) is the most common type of lung cancer. To combat this disease, novel therapeutic targets are badly requirement. ASPM (Abnormal spindle-like microcephaly-associated protein) is involved in multiple cellular or developmental processes, such as neurogenesis and brain growth. ASPM is also reported widely expressed in multiple tumor tissues and involved in the development and progression of several cancers including lung cancer. However, the potential role on ASPM on LSCC is still unclear. In this study, we reported that ASPM was related to the poor prognosis of patients with lung squamous cell carcinoma. Our results further showed that ASPM depletion dramatically inhibited the proliferation of LSCC cells, consistent with the obviously decreased of cyclin D1(CCND1) and cyclin dependent kinases 4 (CDK4) expression. *In vivo* assays further confirmed ASPM ablation markedly blocked tumor growth *in vivo* compared with control. In addition, a co-expression was found between ASPM and CDK4 in human tumor tissues. Taken together, our data provides strong evidence that ASPM promotes lung squamous cell carcinoma proliferation *in vitro* and *in vivo*, and indicates its potential role as a LSCC therapeutic target.

## Introduction

Lung cancer is one of the malignant tumors with the fastest increase in morbidity and mortality and the greatest threat to human health 1,2. The morbidity and mortality of male lung cancer is the highest of all malignant tumors, however, due to its complicated etiology and clinical manifestations, no significant therapeutic progress has been made 3-5. According to different cell sources, lung cancer was mainly divided into lung squamous cell carcinoma (LSCC) and lung adenocarcinoma 6. Among them, LSCC is the most common type of lung cancer 7. Targeted therapy for lung squamous cell carcinoma has made some progress, and some novel discovered therapeutic targets, such as SETD-8 and CDKN2A, have a good prospect of treatment 8,9. However, in order to combat this deadly malignancy, novel therapeutic targets are also badly needed.

Abnormal spindle-like microcephaly-associated protein (ASPM), a type of microtubule associated centrosome protein, plays roles in multiple cellular or developmental processes, including the regulation of neurogenesis and brain growth 10-12. ASPM could maintain symmetric divisions of neuroepithelial cells 13. Additionally, ASPM localizes to the spindle and midbody and is involved in the functional regulation of spindle organization and cytokinesis 14-16. ASPM also controls microtubule disassembly and G1 restriction 17,18.

Recently, ASPM is reported widely expressed in multiple tumor tissues and involved in the development and progression of several cancers 19. ASPM is highly expressed in invasive glioblastoma multiform, and ASPM depletion inhibits the proliferation of glioblastoma cells 20. Furthermore, ASPM has also been reported involved in the progression of diverse tumors, including hepatocellular carcinomas, gastric cancer, pancreatic ductal adenocarcinomas, and lung cancer 21-24. Previous study indicated that ASPM was involved in the development and progression of lung adenocarcinoma and was associated with poor prognosis 24. However, the potential role on ASPM on lung squamous cell carcinoma (LSCC) is still unclear.

Herein, we demonstrated that ASPM was positively associated with the poor prognosis of patients with LSCC. ASPM ablation obviously blocked proliferation *in vitro* and suppressed tumor growth in mice. Thus, ASPM could represent a novel therapeutic target to combat lung squamous cell carcinoma.

## Materials and Methods

### Antibodies, primers and shRNA plasmids

Anti-ASPM (For immunohistochemical analysis, 1:200 dilution, ABIN5913131. For immunoblot, 1:1000 dilution, ABIN960544, Abcam), Anti-β-actin (1:1000 dilution, ab8227, Abcam). Anti-CDK4 (1:1000 dilution for immunoblot, 1:200 dilution for immunohistochemical analysis, ab108357, Abcam), Anti-CCND1 CCND1 (1:10000 dilution, ab134175, Abcam).

The quantitative PCR primer sequences of ASPM were as follows:

- Forward, 5'-GGGAAAGGCAAATGGAAAAC-3'; and,
- Reverse, 5'- CCCAAGGCCATACAAGTGTT-3'.

The quantitative PCR primer sequences of GAPDH were as follows:

- Forward, 5'-CGACCACTTTGTCAAGCTCA-3'; and,
- Reverse, 5'-GGTTGAGCACAGGGTACTTTATT-3'.

The shRNA clone for ASPM was purchased from Addgene, and the targeted sequences of ASPM were as follows: 5'- CCGGTCCTGTCTCTCAGCCACTT-3'.

### Human tissue samples and analysis

A total of 82 human lung squamous cell carcinoma tissues were obtained from the patients receiving surgical treatment in Tianjin medical university cancer institute & hospital. Meanwhile, the clinical pathological characteristics, such as patient ages at diagnosis, genders, tumor stage, tumor size, and recurrence of patients were recorded.

To explore the potential correlations between ASPM expression, CDK4 expression and lung squamous cell carcinoma, immunohistochemical assays were then performed. In brief, sample sections were fixed with 4% PFA at room temperature and subsequently blocked with 2% BSA for 30 minutes at room temperature. Tumor and normal tissue slides were then incubated with anti-ASPM antibody at room temperature for 2 hours. After washed with PBS for 4 times, slides were incubated with biocatalytic secondary antibody for 1.5 hours, and diaminobenzidine was used as a chromogen substrate.

Subsequently, the score criteria were as follows. Briefly, intensity score, 0 means negative staining, 1 represents for weak staining, 2 means modest staining and 3 means strong staining. The proportion of cells with strong staining. For the staining analysis, the staining intensity was multiplied by the proportion of staining cells. Overall score of 5 to 100, 101 to 300 was classified into low-level, and high-level expression, respectively. For the results, ASPM nuclear staining was scored by using 4-point scales (0, no staining; 1+, light staining at high magnification; 2+, intermediate staining; 3+, dark staining of linear membrane at low magnification). According to the distribution of scores, the ASPM was divided into high (2-3+) and low (0-1+) expression groups respectively. Sections that exhibited complete absence of staining or staining in less than 5% of the cells were thought as negative staining.

### Cell culture and transfection

NCI-H520 and SK-MES-1 human LSCC cells were bought from ATCC. Both NCI-H520 and SK-MES-1 cells were cultured in RPMI-1640 and EMEM culture medium, respectively, and supplemented with 10% of fetal bovine serum (FBS). Both cell lines were maintained at 37 °C in a 5% CO_2_ incubator.

The ASPM shRNA plasmids were transfected into LSCC cells by Invitrogen Lipofectamine® 3000 (Thermo Fisher Scientific, Inc.). The specific shRNA above to target ASPM, and scrambled sequence was used as negative control. 100,000 cell per well in six-well plates according to the manufacturer's protocol, 3 groups were set, including: ASPM depletion group, which was transfected with shRNA targeting ASPM; Negative Control group, which was transfected with scrambled sequence; and Mock group was treated without any transfection (data not shown). Silence-efficiency was measured by quantitative PCR and immunoblot after 48 hours' transfection. These reduced cells were used to explore the links between ASPM and cell proliferation and cell invasion. Then the ASPM stable depletion cell lines were screened and used for the *in vitro* and animal assays.

### Quantitative PCR assay

Total RNA was extracted from NCI-H520 and SK-MES-1 cells by Trizol reagent (Invitrogen), respectively. Then the total RNAs was reverse-transcribed by M-MLV reverse transcriptase (Promega). Meanwhile, total RNAs were reverse transcribed to produce cDNA by cDNA synthesis system including dNTP-mix, primer-mix, 5×PrimeScript buffer, DTT and DEPC water. Quantitative PCR was subsequently conducted using SYBR mixture (Takara), and the relative expression level of ASPM was normalized to the expression of GAPDH.

### Western blot assays

Protein samples were extracted from LSCC cells and tumor tissues and the expression of proteins, including ASPM and β-actin was detected through SDS-PAGE. After transmembrane, the PVDF membranes were blocked with 5% dry milk in TBST then incubated with the primary antibodies for the detection of targeted proteins for 2 hours. Then the membranes were incubated with HRP-conjugated secondary antibodies for 45 minutes at room temperature. Signals were visualizal with an ECL kit.

### Cell proliferation assays

For colony formation assay, 1000 cells were added into a 6-well culture plate and transfected with control or ASPM shRNA plasmids and then maintained for 2 weeks. Cells were subsequently fixed with 4% PFA and stained with 0.1% crystal violet at room temperature for 30 minutes and washed with PBS twice. Cell numbers were manually counted.

For MTT assays, LSCC cells were plated in 96-well plates at a density of 500 cells each well, transfected with shRNA plasmids and cultured for 2 days. Cells were then incubated with MTT for 4 hours and removed the culture medium. Subsequently cells were washed with PBS buffer. MTT was extracted by 200 μL DMSO and the absorbance value at a wavelength at 570 nm was detected with a microplate reader.

### *In vivo* tumor growth assays

Nude BalB/c mice (6-8 weeks, 18-22g) were purchased from Beijing Vital River Laboratory Animal Technology Co., Ltd. (Beijing, China). All animal assay procedures were approved by our Institutional Animal Care and Use Committee (IACUC).

Briefly, NCI-H520 cells were infected with control or ASPM shRNA plasmids to stable deplete the expression of ASPM. Approximately 1 x10^ 6^ NCI-H520 cells were implanted into athymic nude mice. 2 weeks later, tumor was isolated, photographed, and the tumor volume was measured every week. Then the tumor growth curves were calculated and compared.

### Statistics

Data were analyzed with SPSS 22.0 software (SPSS Inc, IBM Corp, Armonk, NY). For the immunohistochemistry experiments, associations between ASPM, CDK4 expression and the clinicopathological features were evaluated using χ^2^ tests. Associations of survival and tumor progression and ASPM expression were estimated by Kaplan-Meier method and log-rank tests. Data are shown as the mean ± standard deviation (SD) *in vitro* and animal experiments. Student's t-test was used for statistical comparisons. χ^2^ tests, and correlation analysis (Pearson and Spearman) were performed to analyze the associations between ASPM and CDK4. A value of *P<*0.05 was thought statistically significant.

## Results

### The marked association between the expression of ASPM and the prognosis of patients with lung squamous cell carcinoma

**Figure 1:**
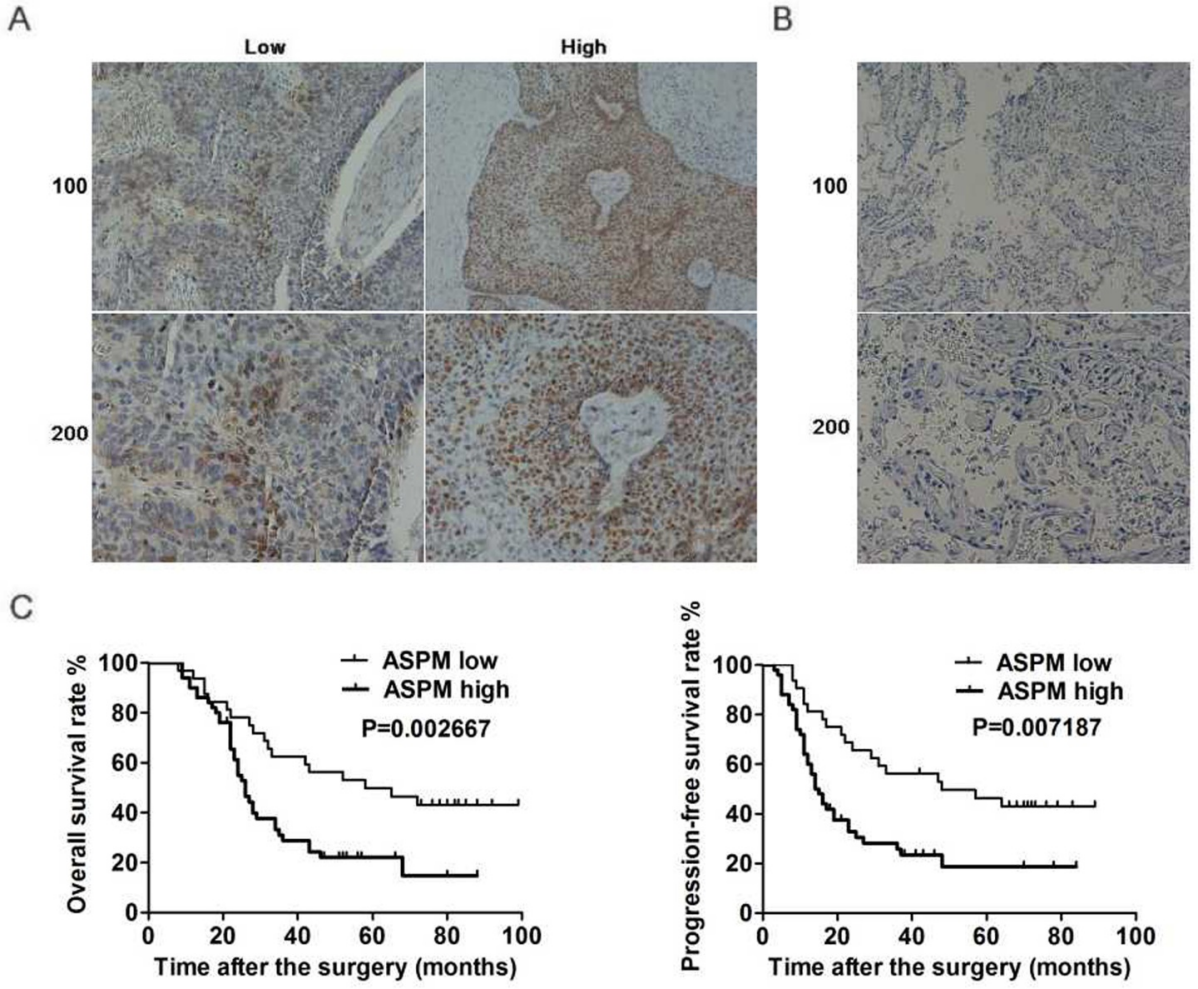
** ASPM expression correlates with the prognosis of LSCC patients.** (**A**) Immunohistochemical assays were performed, and the representive photographs of ASPM expression in LSCC tissues were showed (100× and 200× magnification, respectively). (**B**) Immunohistochemical staining showed ASPM was low expressed in the adjacent tissues (100× and 200× magnification, respectively). (**C**) The KM-Plot analysis of overall survival rate and progression-free survival rate between ASPM low and high expression groups was analyzed and showed.

ASPM, interestingly, was known involved in the progression of mutiple tumors. Furthermore, ASPM was also reported to be associated with the poor prognosis of patients with lung adenocarcinoma. Thereby, to explore the potential function of ASPM in the development of lung squamous cell carcinoma, another large type of lung cancer, several assays were all performed. Firstly, ASPM expression in tumor tissues of lung squamous cell carcinoma patients who underwent surgical resection was examined by immunohistochemistry assays. 82 surgical samples were divided into two groups according to the staining intensity of ASPM (Figure 1A). Data showed that 50 samples belonged to ASPM high-expression while others were low-expression (Table 1). And the corresponding non-tumor normal tissues showed significant low expression of ASPM (Figure 1B).

Clinicopathological characteristic of patients with lung squamous cell carcinoma was then analyzed between ASPM low and high-expression groups. Patient gender, smoking, drinking, tumor grade and lymph node metastasis, etc. was recorded, respectively. According to the analysis results, no significant difference was found in these respects between ASPM low and high expression groups (Table 1). Whereas the expression level of ASPM in tumor tissues was obviously related to tumor size (*P*=0.024) and clinical stage (*P*=0.021) (Table 1).

On this basis, we performed Kaplan-Meier analysis assay to explore the link between ASPM expression and prognosis of patients with lung squamous cell carcinoma. Results showed that ASPM expression was correlated with overall survival (OS) rate and progression-free survival (PFS) rate (Figure 1C). In conclusion, these data indicated a significant link between ASPM and the prognosis of patients with lung squamous cell carcinoma.

### ASPM promotes lung squamous cell carcinoma proliferation *in vitro*

**Figure 2:**
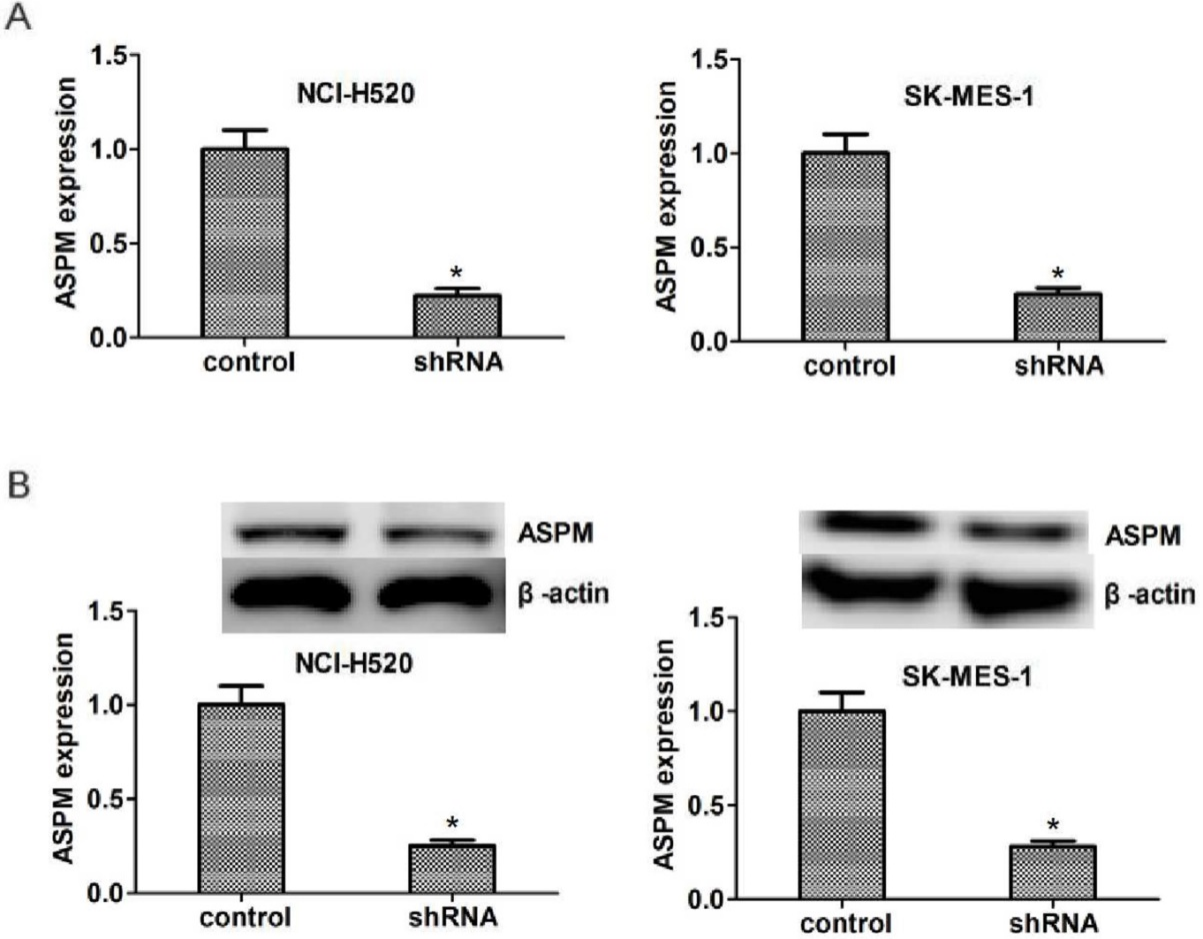
ASPM expression was effectively decreased in both NCI-H520 and SK-MES-1 cells caused by the transfection of ASPM shRNA. (**A**) qPCR assays revealed the significantly dropped expression level of ASPM in ASPM shRNA-treated NCI-H520 and SK-MES-1 cells, respectively. (**B**) Immunoblot assays confirmed the efficiently silenced of ASPM expression caused by its shRNA in both NCI-H520 and SK-MES-1 cells. Results are presented as mean ± SD, * P < 0.05.

To explore the potential regulatory mechanism of ASPM in the progression of lung squamous cell carcinoma, the shRNA specifically targeted ASPM was transfected into two types of LSCC cells, NCI-H520 and SK-MES-1 cells, to knockdown the expression of ASPM. Quantitative PCR (Figure 2A) and westernblot (Figure 2B) assays showed the shRNA of ASPM effectively inhibited its expression level in both NCI-H520 and SK-MES-1 cells, respectively.

**Figure 3:**
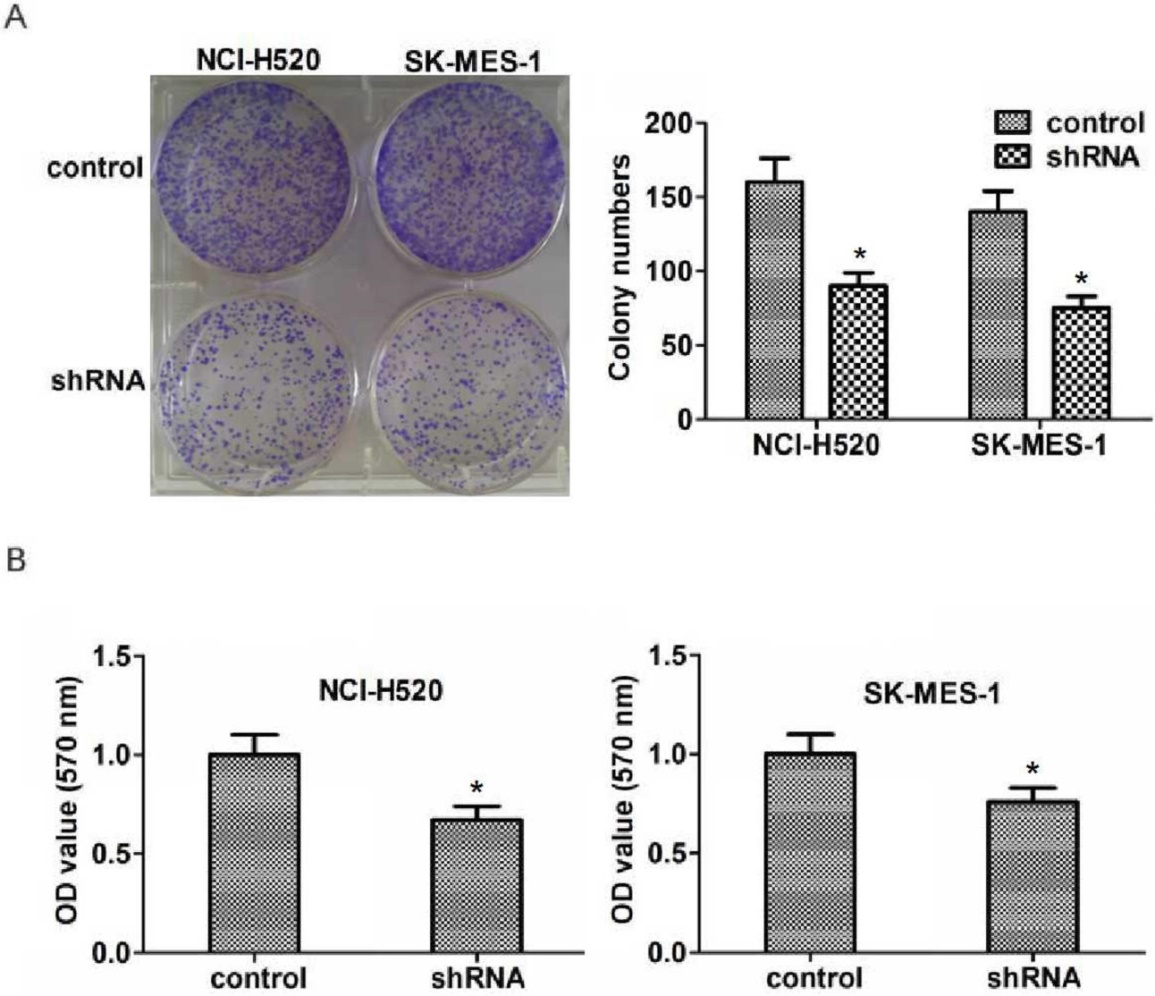
** ASPM promotes the proliferation of LSCC cells *in vitro*.** (**A**). Representive photographs showed the results of colony formation assays of NCI-H520 and SK-MES-1 cells transfected with control or ASPM shRNA. (**B**) MTT assays showed the decrease of cell proliferation capacity caused by ASPM depletion.

Abnormal cell proliferation is a key factor in the development and progression of tumors. To explore the potential role of ASPM in the proliferation of lung squamous cell carcinoma, colony formation assays were then performed. As expected, we found that knockdown of ASPM dramatically blocked the proliferation of NCI-H520 and SK-MES-1 cells (Figure 3A). Similarly, a markedly dropped absorbance value at 570 nm wavelength in NCI-H520 and SK-MES-1 cells was measured through performing MTT assays (Figure 3B).

**Figure 4:**
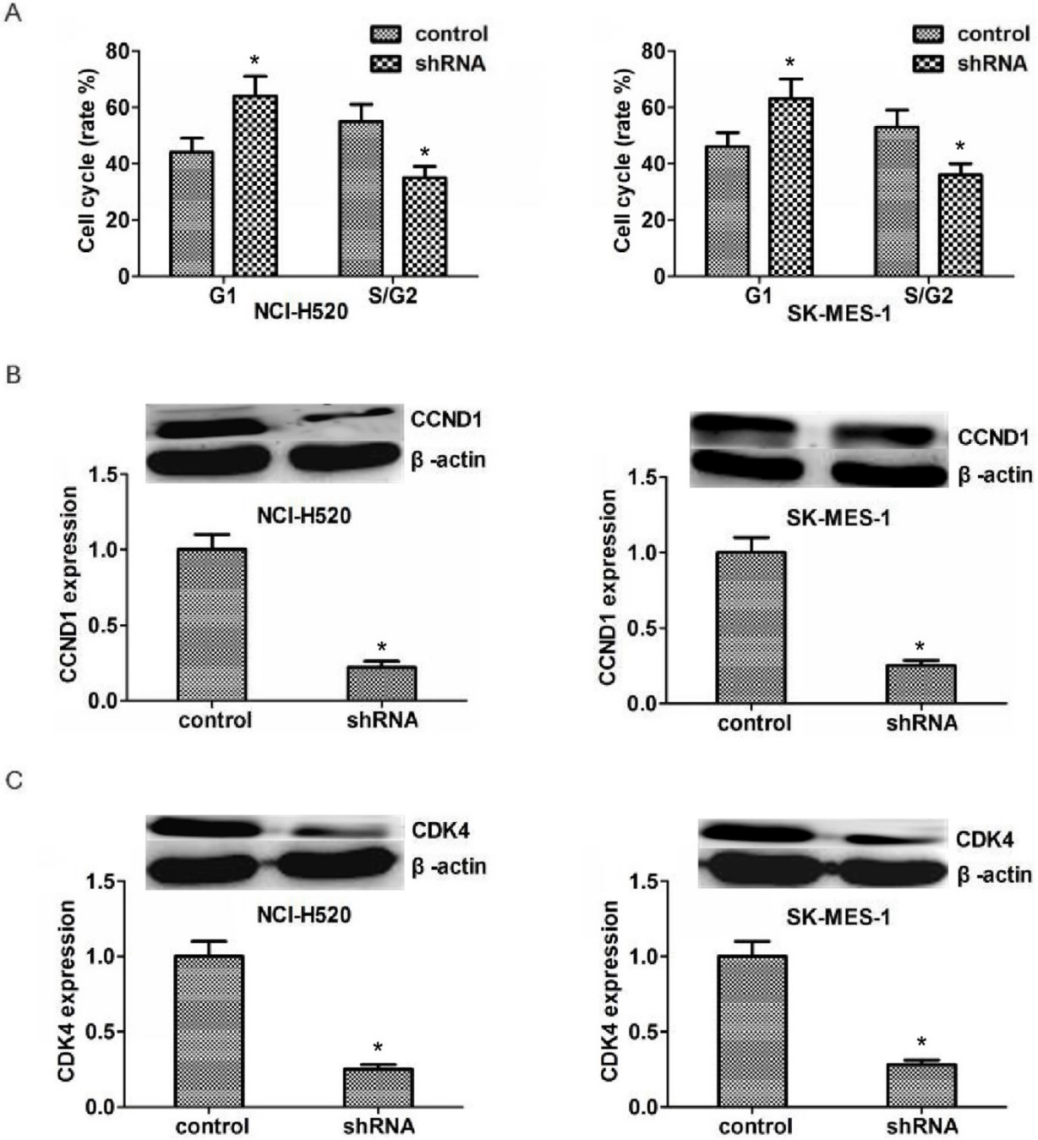
** ASPM depletion affected the cell cycle by inhibiting the expression of CCND1 and CDK4.** (**A**) ASPM depletion resulted in an obvious increase of G1 stage and an obvious decrease of S/G2stage. (**B**).Western blot assays revealed the significantly dropped expression level of CCND1 in ASPM shRNA-treated NCI-H520 and SK-MES-1 cells. (**C**).Western blot assays showed the obvious decrease expression level of CDK4 caused by ASPM depletion in both NCI-H520 and SK-MES-1 cells, repectively. Results are presented as mean ± SD, * P < 0.05.

Since ASPM could significantly affect the proliferation capacity of LSCC, we hypothesize that it might affect proliferation through the regulation of cell cycle. And our results proved that ASPM depletion resulted in an obvious increase of G1 stage and an obvious decrease of S/G2stage (Figure 4A). We tested the transcription levels of CCND1 and CDK4, and found that in both NCI-H520 and SK-MES-1 cells, ASPM depletion resulted in an obvious decrease of the CCND1 and CDK4 protein level, respectively (Figure 4B and 4C).

Taken together, data indicated the involvement of ASPM in the regulation of LSCC cell proliferation *in vitro*.

### ASPM promotes lung squamous cell carcinoma progression in mice

According to the previous data, ASPM ablation led to the defect of proliferation capacity of LSCC cells, we then explored the potential function of ASPM in the growth and progression of LSCC *in vivo*.

**Figure 5:**
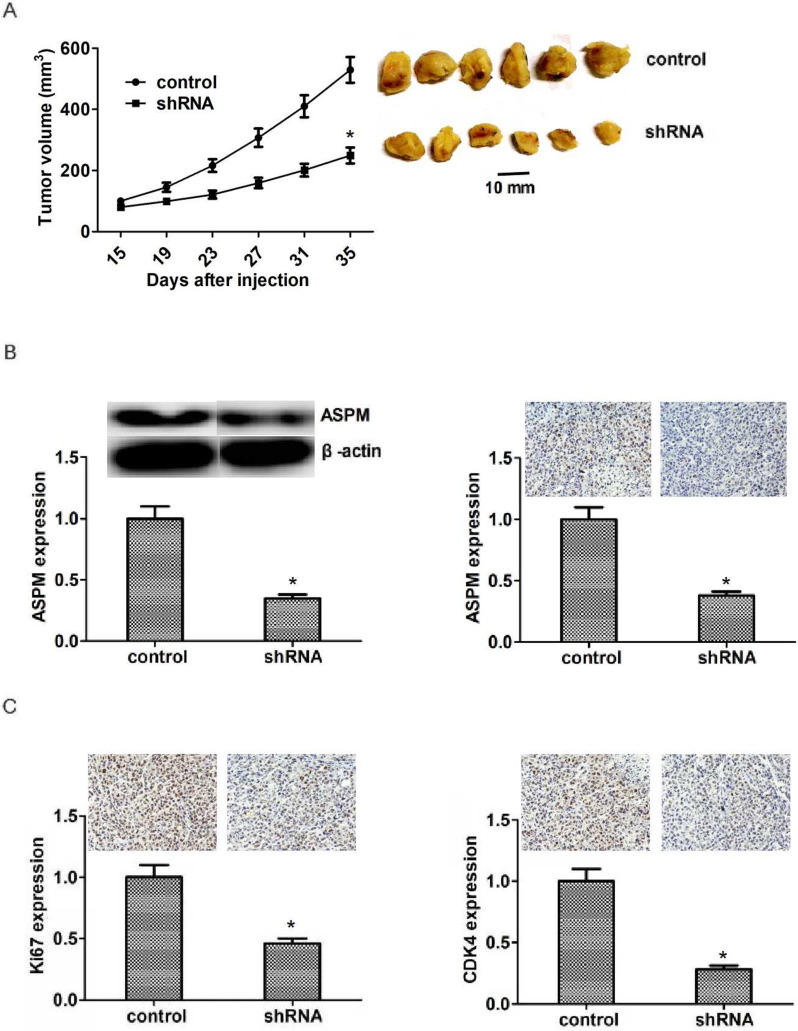
** ASPM ablation impaired LSCC tumor growth in mice.** (**A**) NCI-H520 cells infected with ASPM or control shRNA lentivirus were implanted into nude mice. 2 weeks later, tumors were isolated, and volume was calculated each week. (n=8 in each group). Tumor growth curves were calculated and analyzed according to the average volume of 6 tumors for each group. (**B**). Immunoblot assays and immunohistochemical assays showed the expression level of ASPM in both control or ASPM ablation tumor tissues isolated from mice. (**C**). Immunohistochemical assays indicated the Ki67 and CDK4 expression in control or ASPM knockdown tumor tissue. Results are presented as mean ± SD, * P < 0.05.

To perform the *in vivo* assay, NCI-H520 cells were infected with control or ASPM shRNA lentivirus and subsequently injected into nude mice. After two weeks, tumors were the formation, and the volume was detected every week. Representative photos of tumors were taken and showed in Figure 5A. Interestingly, the volume of tumors isolated from ASPM knockdown groups was obviously smaller than that in control (Figure 5A). Additionally, we performed Immunoblot assay to detect the expression level of ASPM in both control and ASPM depletion tumors and found that the expression level of ASPM was remarkably decreased in ASPM depletion groups, compared with control (Figure 5B). Similarly, immunohistochemistry assay confirmed that the expression level of Ki67 or CDK4 was remarkably decreased in ASPM depletion groups, compared with control (Figure 5C).

Taken together, these results revealed that ASPM promotes the progression of LSCC *in vivo*.

### Co-expression existed between ASPM and CDK4 in LSCC tissues

**Figure 6:**
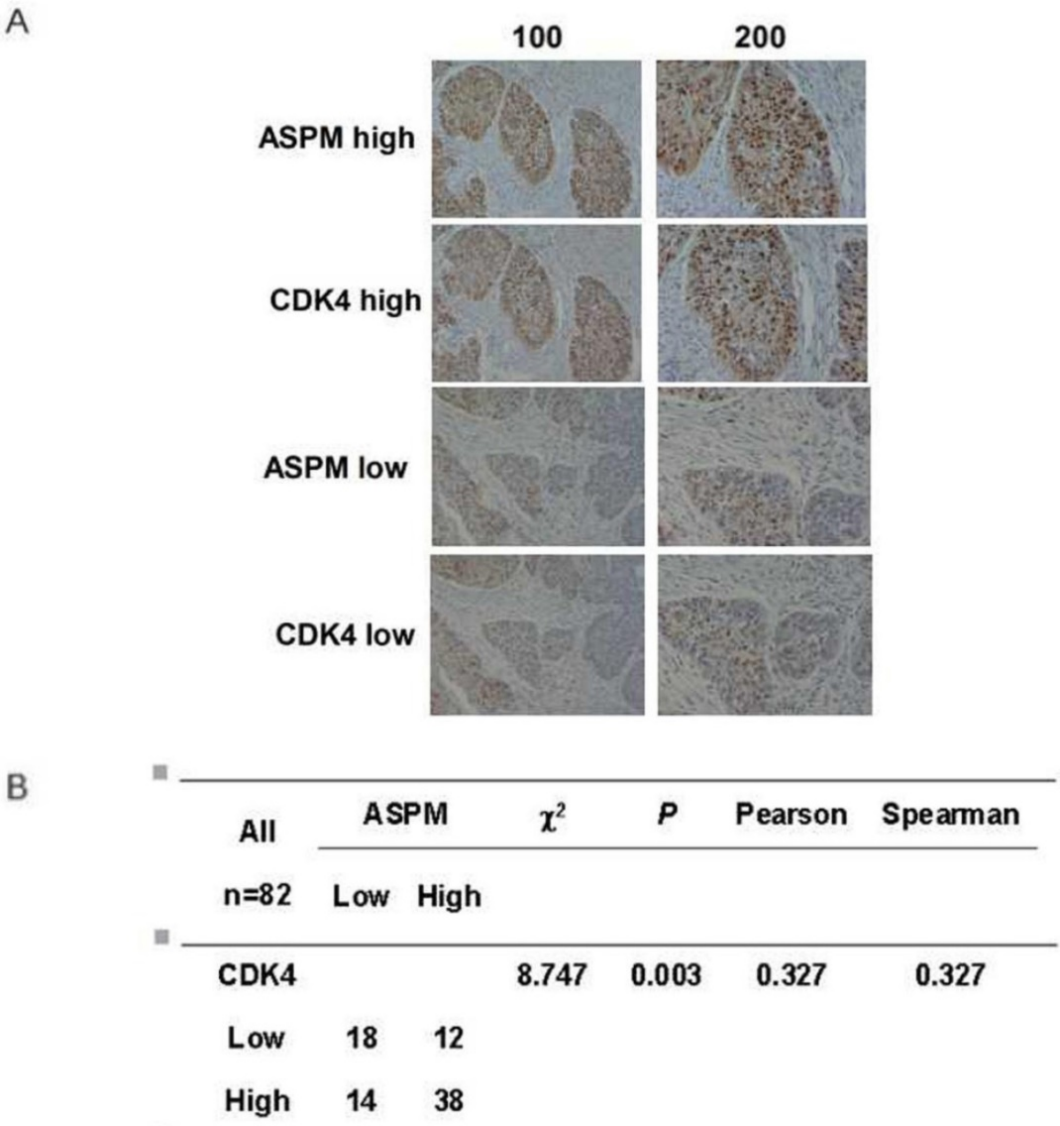
** Co-expression between ASPM and CDK4 in tumor tissues by immunohistochemistry.** (**A**) The immunohistochemistry of ASPM and CDK4 in 82 samples were performed. The typical strong and weak staining was shown. (**B**) χ^2^ tests, and correlation analysis (Pearson and Spearman) were performed to analyze the associations between ASPM and CDK4. The results found that an obvious positive correlation was existed (P=0.003<0.05).

As ASPM affects cell cycle and the related proteins, the relationship between ASPM and CDK4 can be explored in human LSCC tissues. In our study, we used the immunohistochemistry to observe the association between the expression of ASPM and CDK4 in tumor tissue by uninterrupted slicing. The typical staining was shown (Figure 6A). The results found that an obvious positive correlation was existed (Figure 6B). Therefore, we speculated that ASPM possibly played roles in LSCC through regulating the expression of CDK4 and relevant pathways.

## Discussion

Lung cancer is a malignant tumor with high morbidity and mortality 25. Since the progress of traditional medical technology and the emergence of new treatment methods, such as targeted therapy and immunotherapy, the prognosis and five-year survival rate of various tumors are significantly improved 26. Whereas the mortality of lung cancer is always high, due to the lack of obvious early symptoms, high heterogeneity and high metastasis 27. Lung squamous cell carcinoma is the main type of lung cancer, which has a variety of molecular types, and drugs targeted at traditional therapeutic targets, such as Iressa, Trocar and Kemena, have made some progress in the treatment of LSCC as well as improved the survival rate of LSCC patients, however, to further improve the prognosis, novel therapeutic targets are badly needed 28. Herein, we found that ASPM, a centrosome protein, was associated with the poor prognosis of LSCC patients, suggesting that it could be a novel therapeutic target for LSCC. More detailed molecular mechanisms underlying ASPM promote LSCC require further study.

It is known that ASPM is widely expressed in different tissues and affects the occurrence and development of multiple types of tumors 19. The expression of ASPM was associated with the poor prognosis of glioma and was high-expressed in recurrent tumors 29. Additionally, ASPM was involved in the progression of ovarian cancer, and associated with the clinical features, such as tumor grade, of ovarian cancer 30. ASPM high-expression was found in tumor tissues of hepatocellular carcinomas, an observation correlated with increased invasive capacity and high tumor recurrence 31. ASPM high-expression correlated with poor patient survival was also identified in pancreatic cancer 23. Previous studies have suggested that ASPM may be a potential target for the treatment of lung adenocarcinoma through screening, also, ASPM gene was frequently altered by missense and nonsense mutations 24. Interestingly, we reported that ASPM promoted the proliferation of LSCC *in vitro* and *in vivo*, and further found that ASPM expression was associated with tumor size and clinical stage, indicating a tight link between ASPM and LSCC. Whether ASPM plays a similar role in lung adenocarcinoma deserves further study.

As cell cycle regulators, CCND1 and CDK4 play critical roles in regulating the cell cycle of normal and tumor cells 32. ASPM deletion or down-regulation could lead to cell cycle arrest at G1/S phase, thereby inhibiting cell proliferation. Abnormal high expression of CCND1 or CDK4 has been found in various tumors, such as pancreatic cancer, bladder cancer and gastric cancer 32. In addition, many proto-oncogenes could regulate the CCND1/CDK4 signaling pathway, thereby promoting the abnormal proliferation of tumor cells through cell cycle 32. Herein, we found that ASPM ablation reduced the expression levels of CCND1 and CDK4, which may cause the disorder of LSCC cell cycle and further block cell proliferation and tumor development. However, why ASPM knockdown leads to significant down-regulation of CCND1 and CDK4 is worth further study.

As we know, ASPM, which is located at the spindle poles in metaphase of mitotic, is involved in the organization and orientation of spindles, and its deletion or mutation could lead to the disorder of cell cycle 14. ASPM could regulate the stability of cyclin E, thus affecting mitosis duration: ASPM has been shown to regulate the activity of Cyclin E/Cdk2 complex by stabilizing the Cyclin E protein 18. However, in human LSCC, we found that ASPM ablation resulted in a dramatical dropped of the expression level of CCND1 and CDK4, which would lead to a G1/S phage block. Moreover, in tumor tissue by uninterrupted slicing and IHC assay we found that an obvious positive correlationbetween ASPM and CDK4 but not CCND1 (data not shown) was existed. The relationship between ASPM and CDK4 or CCND1 and the related mechanism should be further explored, and perhaps the role and the mechanism are different in different kinds of cancers. Additionally, previous studies indicated that ASPM regulated microtubule disassembly at spindle poles by mediating microtubule dynamics 17. ASPM was also involved in DNA double-strand break repair through a non-homologous end joining manner 33. Collectively, these evidence strongly suggests that ASPM plays an important role in regulating cell proliferation. In fact, ASPM depletion blocks neural stem cell and tumor cell proliferation, thereby affects neurogenesis and tumor growth 34. Studies in animal model indicated that ASPM was involved in brain growth, partly caused by the regulation of proliferation 35. Several studies have shown that ASPM can regulate the proliferation of tumor cells to affect the occurrence and development of tumors 36. In this study, we found that ASPM promoted the progression of LSCC through a proliferation-dependent manner, further confirmed that ASPM regulated proliferation through different molecular mechanisms and thus affected a variety of physiological and pathological processes.

## Figures and Tables

**Table 1:** Relationships of ASPM and clinicopathological characteristics in 82 patients with lung squamous cell carcinoma (*P < 0.05.)

Feature	All n=82	ASPM expression		χ*^2^*	*P*
		Low	High		
		n=32	n=50		
**Age (year)**				1.333	0.248
< 55	50	22	28		
≥ 55	32	10	22		
**Gender**				0.504	0.478
Male	45	16	29		
Female	37	16	21		
**Smoking**				1.339	0.247
Yes	62	22	40		
No	20	10	10		
**Tumor size**				5.101	0.024*
< 4 cm	36	19	17		
≥ 4 cm	46	13	33		
**Differentiation**				1.718	0.190
Low	24	12	12		
High	58	20	38		
**Clinical stage**				5.316	0.021*
I	24	14	10		
II-III	58	18	40		
**Lymphnode metastasis**				0.971	0.324
Yes	38	17	21		
No	44	15	29		

## Abbreviations

ASPM: Abnormal spindle-like microcephaly-associated protein
LSCC: Lung squamous cell carcinoma
CCND1: Cyclin D1
CDK4: Cyclin dependent kinases 4
OS: Overall survival
PFS: Progression-free survival
IHC: Immunohistochemical
DAB: 3,3-diaminobenzidin
HRP: Horseradish peroxidase
PCNA: Proliferating cell nuclear antigen
PBS: Phosphate-buffered saline
PAGE: Polyacrylamide gel electrophoresis
SD: Standard deviation
QRT-PCR: Quantificational real-time polymerase chain reaction
