# Abnormal spindle-like microcephaly-associated protein (ASPM) contributes to the progression of Lung Squamous Cell Carcinoma (LSCC) by regulating CDK4: Erratum

**DOI:** 10.7150/jca.88770

**Published:** 2023-09-06

**Authors:** Ya-Jing Yuan, Yao Sun, Rong Gao, Zhen-zhen Yin, Zhi-yong Yuan, Li-Ming Xu

**Affiliations:** 1 Department of Anesthesia, Tianjin medical university cancer institute & hospital, National clinical research center for cancer, Key laboratory of cancer prevention and therapy, Tianjin's Clinical Research Center for Cancer, Tianjin, 300060, China.; 2 Department of Radiation Oncology, Tianjin medical university cancer institute & hospital, National clinical research center for cancer, Key laboratory of Cancer Prevention and Therapy, Tianjin's clinical research center for cancer, Tianjin, 300060, China.; 3 Department of Pathology, Gansu Medical College, Pingliang City, Gansu Province, 744000, China.; 4 Department of Radiation Oncology, Tianjin Medical University Cancer Hospital airport hospital, Tianjin, 300308, China.

Due to a typographical error by our team members, the layout of Figure 5 was duplicated. We repeated experiments to validate the Figure 2B, Figure 3A and Figure 4B-C. Now we confirm that we have preserved Western blot, the six-well plates of clonal formation experiments and the wax lumps of tumor tissue for the objective truth and unique subordination of data. This correction does not affect the results and conclusions. We apologize for the errors and the inconveniences caused by our mistake.

All authors confirm that they agree to the erratum.

## Figures and Tables

**Figure 2:**
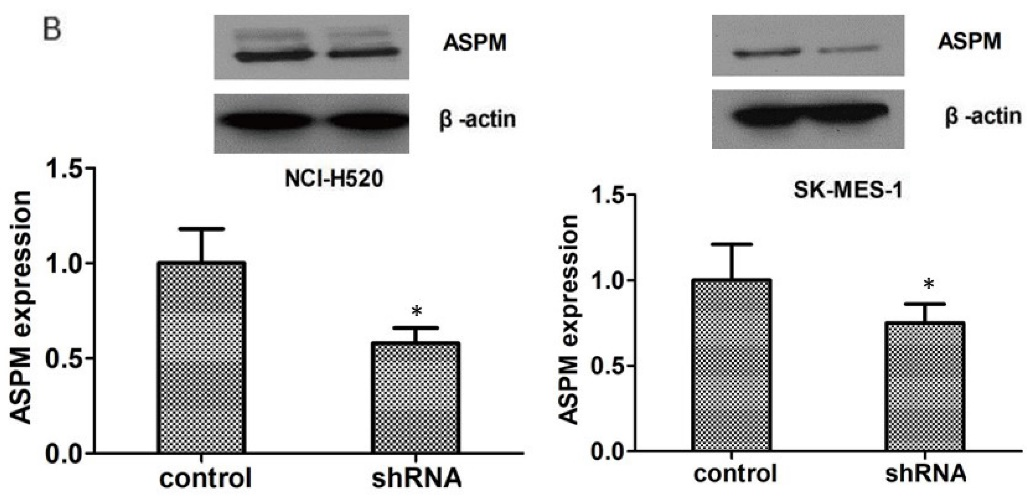
(B) Immunoblot assays confirmed the efficiently silenced of ASPM expression caused by its shRNA in both NCI-H520 and SK-MES-1 cells. Results are presented as mean ± SD, * P < 0.0

**Figure 3:**
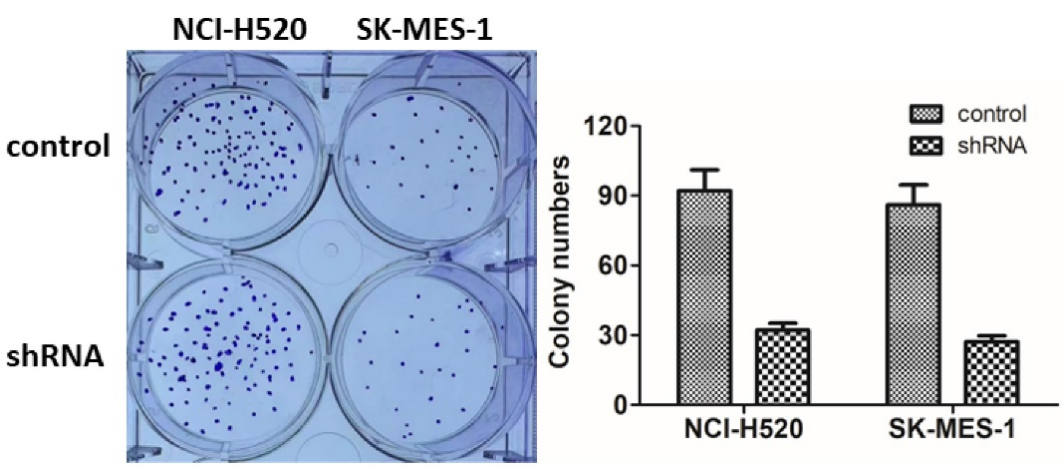
** ASPM promotes the proliferation of LSCC cells in vitro.** (A). Representative photographs showed the results of colony formation assays of NCI-H520 and SK-MES-1 cells transfected with control or ASPM shRNA.

**Figure 4:**
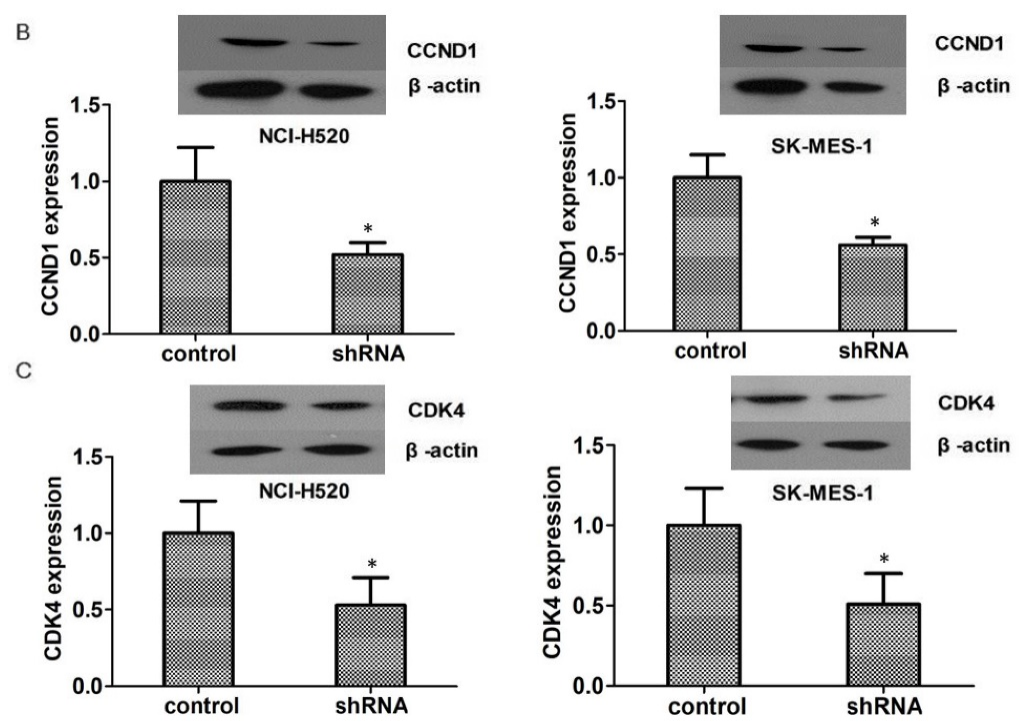
(B). Western blot assays revealed the significantly dropped expression level of CCND1 in ASPM shRNA-treated NCI-H520 and SK-MES-1 cells. (C). Western blot assays showed the obvious decrease expression level of CDK4 caused by ASPM depletion in both NCI-H520 and SK-MES-1 cells, respectively. Results are presented as mean ± SD, * P < 0.05.

**Figure 5:**
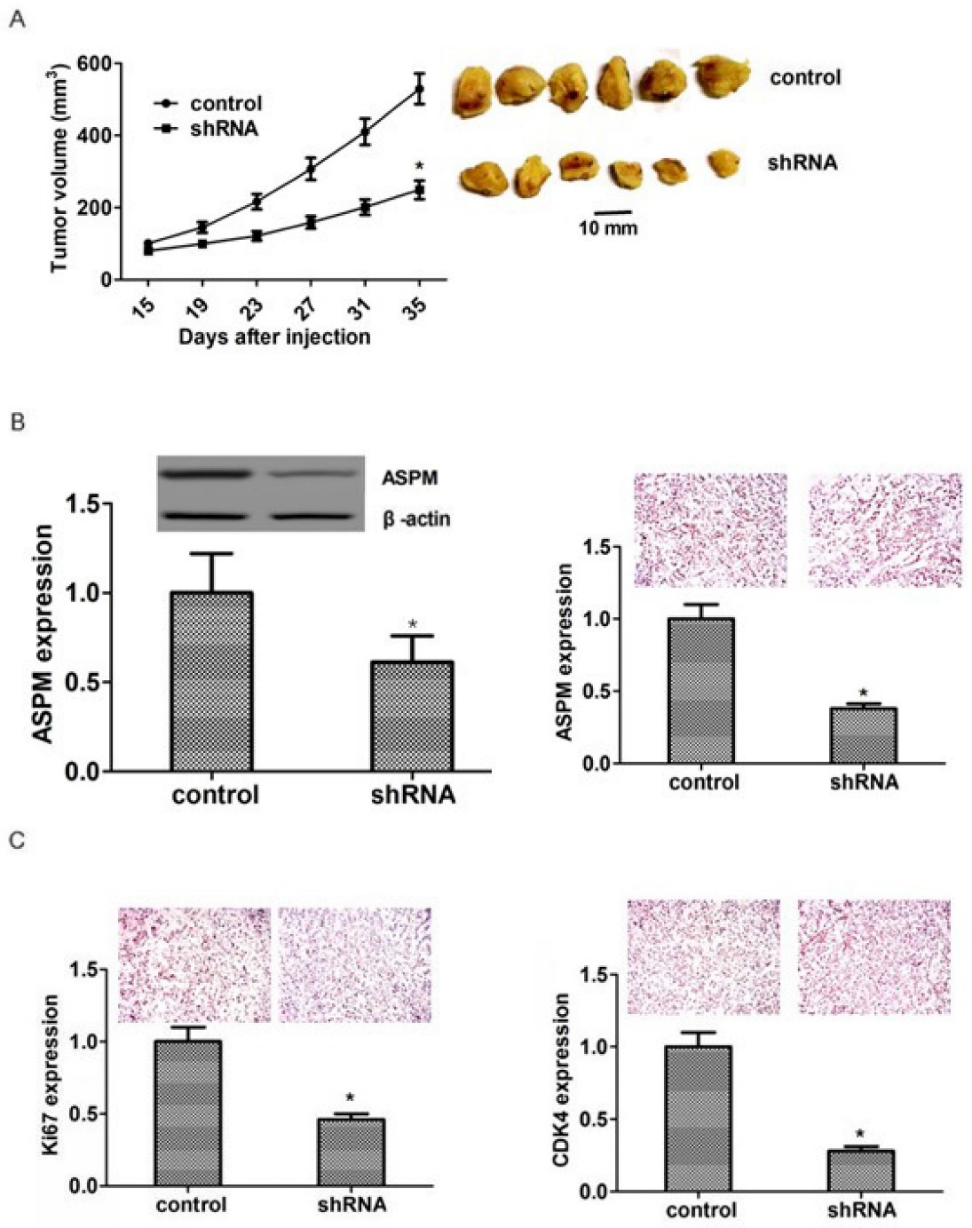
** ASPM ablation impaired LSCC tumor growth in mice.** (A) NCI-H520 cells infected with ASPM or control shRNA lentivirus were implanted into nude mice. 2 weeks later, tumors were isolated, and volume was calculated each week. (n=8 in each group). Tumor growth curves were calculated and analyzed according to the average volume of 6 tumors for each group. (B). Immunoblot assays and immunohistochemical assays showed the expression level of ASPM in both control or ASPM ablation tumor tissues isolated from mice. (C). Immunohistochemical assays indicated the Ki67 and CDK4 expression in control or ASPM knockdown tumor tissue. Results are presented as mean ± SD, * P < 0.05.

